# DIGITAL LEARNING AND ONLINE CHAIR YOGA FOR RURAL UNDERSERVED OLDER ADULTS AT RISK OF COGNITIVE DECLINE

**DOI:** 10.1093/geroni/igac059.372

**Published:** 2022-12-20

**Authors:** Lisa Wiese, JuYoung Park

**Affiliations:** Florida Atlantic University, Boca Raton, Florida, United States; Florida Atlantic University College of Social Work and Criminal Justice, Boca Raton, Florida, United States

## Abstract

We conducted a randomized control trial to test the feasibility of an online chair yoga intervention among rural older adults at risk for cognitive decline in an underserved, racially/ethnically diverse community. Participants were randomly assigned to either chair yoga (n=15) or computer brain games (n=15). Prior to the 12-week intervention, a computer training program was provided to all participants by local high school students, who followed a previously tested curriculum, and mentored the older adults in attending sessions. Digital literacy and cognitive, physical, and psychosocial measures were collected at baseline, 3 weeks, and post-intervention, with 3 and 6-month follow-up. We assessed students’ attitudes toward older adults pre/post-intervention. Both student and resident samples were 98% minority (African American, Latino, and Afro-Caribbean), with residents’ average age of 67.5 (SD = 8.3), years lived rural 38 (M = 11.5), and initial digital literacy of 48.5%. Additional outcomes will be detailed in this presentation.

